# Potential Value of Triple Drug Therapy with Ivermectin, Diethylcarbamazine, and Albendazole (IDA) to Accelerate Elimination of Lymphatic Filariasis and Onchocerciasis in Africa

**DOI:** 10.1371/journal.pntd.0005163

**Published:** 2017-01-05

**Authors:** Peter U. Fischer, Christopher L. King, Julie A. Jacobson, Gary J. Weil

**Affiliations:** 1 Infectious Diseases Division, Department of Internal Medicine, Washington University School of Medicine, St. Louis, Missouri, United States of America; 2 Case Western Reserve University, Cleveland, Ohio, United States of America; 3 Bill and Melinda Gates Foundation, Seattle, Washington, United States of America; McGill University, CANADA

## Overview

Recent studies have shown that single-dose combination therapy with three currently approved antifilarial drugs (ivermectin, diethylcarbamazine (DEC), and albendazole, or IDA) is superior to current regimens used in lymphatic filariasis (LF) elimination programs. IDA may help to accelerate LF elimination in Africa, which has lagged behind other regions. Although it has not yet been tested, IDA may also be useful for treating onchocerciasis. There is a serious concern about using DEC in sub-Saharan Africa because of ocular adverse events after DEC treatment of onchocerciasis in the past. This paper discusses published experience with DEC in onchocerciasis patients and describes strategies for studying the effects of IDA in patients with onchocerciasis and LF in Africa.

## Introduction

The Global Program to Eliminate Lymphatic Filariasis (GPELF) has made significant progress in many countries. GPELF has delivered 5,600 million treatments to more than 763 million people living in 61 countries between the years 2000 and 2014, and it was estimated to have prevented 36 million clinical cases and saved 175 million disability adjusted life years (DALYs) [1]. Although elimination of LF is a major goal of the London Declaration on Neglected Tropical Diseases (NTDs), it is unlikely that LF will be eliminated by the target year of 2020 [2]. The key strategy of the elimination program comprises repeated annual rounds of mass drug administration (MDA) to populations at risk of acquiring the infection with diethylcarbamazine (DEC) plus albendazole outside of Africa and with ivermectin plus albendazole in Africa [3]. MDA reduces microfilariae (Mf) in human blood that are necessary for transmission by mosquito vectors, and one aim of the program is to interrupt transmission. Repeated rounds of MDA are required because current regimens fail to kill all adult worms and completely clear Mf following single-dose treatments. Successful programs have sustained annual MDA with high compliance for several years. Seventy-three countries were considered to be endemic for LF in 2015, and 55 of them still required MDA [4]. The GPELF has lagged in sub-Saharan Africa, where only 2 of 35 LF-endemic countries have stopped MDA and started post-MDA surveillance. Many countries in this region have either not started MDA or have less than 65% geographical coverage. Hence, a more effective treatment strategy could have a major impact on LF elimination in Africa.

The goal of onchocerciasis intervention in Africa has also shifted in recent years from control towards elimination [5, 6]. As with LF, the key strategy for onchocerciasis intervention is MDA. In most countries, this is accomplished with annual community-directed treatment with ivermectin (CDTi). Modeling studies predict that current strategies will permit many countries to reach targets for stopping MDA for onchocerciasis by 2028, but intervention is likely to be required for a much longer period in some areas (especially in Central Africa) [7]. While annual or semiannual CDTi for 15 to 17 years eliminated the infection in some areas, it was not sufficient to stop transmission in others [8, 9]. Therefore, extensive research is currently ongoing to identify drugs that are more effective than ivermectin. Drug development studies are time and resource intensive, and it is unlikely that a novel drug for onchocerciasis will be registered to significantly advance the progress of onchocerciasis elimination within the next 5 years.

In this paper, we discuss the potential value of combination therapy with three currently approved drugs (ivermectin, DEC, and albendazole, or IDA) for advancing LF and onchocerciasis elimination programs in Africa. Because DEC treatment can cause ocular adverse events in persons with heavy *Onchocerca volvulus* infections, studies of the new combination will have to be conducted with proper precautions. Therefore, this paper also discusses prior experience with DEC in onchocerciasis patients and a strategy for studying the effects of IDA in patients with onchocerciasis.

## Ivermectin, DEC, and Albendazole (IDA) Treatment for Lymphatic Filariasis

Thomsen et al. have recently reported that a single dose of coadministered IDA was superior to the standard two-drug regimen of DEC with albendazole for clearing *Wuchereria bancrofti* Mf in infected subjects in Papua New Guinea [10]. All 12 of the heavily infected subjects treated with IDA had complete clearance of Mf one year after treatment, whereas only 1 of 12 subjects treated with DEC plus albendazole had complete clearance of Mf at 12 months. In addition, all 6 IDA-treated subjects retested at 24 months were still Mf negative at that time. This suggests that a single dose of IDA either kills or permanently sterilizes adult *W*. *bancrofti* worms and prevents their hosts from contributing parasites for transmission by mosquitoes. The Thomsen et al. study also showed that IDA was safe and that there were no significant interactions between the drugs in the IDA regimen. Early results from larger clinical trials of IDA in Papua New Guinea and Cote d’Ivoire are consistent with the results reported by Thomsen et al. These results suggest that IDA has the potential to dramatically accelerate LF elimination programs. Several large community trials are underway to confirm the safety and efficacy of IDA in different epidemiological settings. Because DEC has the potential to cause ocular adverse events in individuals infected with *O*. *volvulus*, all of these studies are being conducted in areas without coendemic onchocerciasis.

## IDA for LF Elimination in Areas Not Coendemic for Onchocerciasis

Although most sub-Saharan countries endemic for LF use ivermectin combined with albendazole in their elimination programs, the region includes also countries or areas that are nonendemic for onchocerciasis or loiasis (e.g., Kenya, Zanzibar, Comoros, Sao Tome and Principe, Eritrea, Madagascar, Zambia, and Zimbabwe), and these countries currently use or plan to use DEC combined with albendazole in their LF elimination programs [11]. These countries stand to immediately benefit from the more effective IDA regimen if results from ongoing safety trials are favorable. For other countries, we need to consider how IDA might be used for LF elimination in areas without coendemic onchocerciasis. In some countries (e.g., Tanzania) onchocerciasis foci are limited and geographically separated from LF-endemic areas, and IDA might be used to eliminate LF in these areas at the district level. However, this strategy requires accurate disease maps, careful assessment of human migration patterns, an ability to safely screen out individuals that may be at risk of onchocerciasis, and an understanding of the effects and adverse events associated with DEC treatment of onchocerciasis.

## Efficacy and Safety of DEC for Treatment of Onchocerciasis

Diethylcarbamazine (N, N-diethyl-4-methyl-1-piperazine carboxamide) was shown to be effective against filarial parasites in the late 1940s [12–15]. Although it was recognized early that DEC can cause adverse reactions in individuals infected with *O*. *volvulus*, it was the recommended first line treatment for onchocerciasis for about 40 years because better alternatives were not available until ivermectin was introduced [16–18]. A treatment course of oral DEC was used for both individual and community treatment [19]. In addition, topical DEC or eye drops containing DEC were sometimes used to specifically target skin or ocular disease, respectively [20–22]. Since eye lesions were among the most severe clinical signs of *O*. *volvulus* infection, people with ocular involvement were often preferentially treated [23]. DEC was administered in multiple doses, and many different dosing regimens were evaluated (Table 1). One common regimen recommended for a 50 kg adult was a total dose of 1,350 mg over eight days (average total dose per course of 27 mg/kg body weight), with a low dose of 1 mg/kg on day one and higher doses thereafter. Patients were often treated with a second course of DEC four months later [16]. The efficacy and safety of single-dose treatment with DEC (6 mg/kg) combined with albendazole (400 mg) used for elimination of LF outside Africa was never evaluated in patients with onchocerciasis because of the risk of inducing Mazzotti reactions and ocular adverse events in Mf-positive individuals and because ivermectin was much better tolerated. DEC-fortified cooking salt that is sometimes used for LF intervention was reported to be safe but not effective in subjects with onchocerciasis [24].

**Table 1:** Selected studies that report adverse events of onchocerciasis patients after treatment with DEC compared with a potential study design of IDA.

Location/Type of the study	Participant characteristics	DEC dosing	Adverse events in DEC group	Reference
Liberia/randomized clinical trial (RCT) oral/topical	Ten men; mean Mf 7 Mf/mg skin; no obvious eye disease	1,100 mg over eight days, ~5,450 mg over six months, total about 109 mg/kg	Initial Mazzotti* reaction; Fluffy corneal opacities in nine patients first detected seven days after beginning of treatment, other ocular changes, no follow-up after conclusion of treatment	[28]
Southern Sudan/observational	18 men; >50 Mf/mg; no acute eye disease, but ocular Mf	925 mg over eight days, total ~15 mg/kg	Mazzotti reaction in all patients; new ocular lesions (some transient, some sustained) in most patients starting two days after beginning of treatment, no assessment after treatment	[26]
Liberia/randomized observational	Ten men; low to moderate skin Mf	1,100 mg over eight days, ~5,450 mg over six months, total about 109 mg/kg	Initial Mazzotti reaction; Anterior uveitis in most patients, some patients with chorioretinitis, optic nerve pallor, visual field constriction, adverse events associated with circulating immune complexes, no follow-up after conclusion of treatment	[33]
Northern Ghana/observational	21 men; varying skin Mf density, no eye assessments	200 mg/day for seven days, total ~23 mg/kg	Mazzotti reaction, severe pruritus, lymphadenopathy, and hypotension related to Mf density	[32]
Mali/placebo-controlled RCT with ivermectin arm	Ten men; ~100 Mf/mg; 70% punctate keratitis, 50% Mf in the anterior chamber	1,300 mg over eight days, total ~22 mg/kg	50% with mild to moderate Mazzotti reaction, 50% with severe Mazzotti; ocular findings unchanged, only one patient with new ocular lesions at six months	[51]
Liberia/placebo-controlled RCT with ivermectin arm	Ten men; ~ 40 mf/mg; 90% with eye disease and/or ocular Mf	1,300 mg over eight days, total ~22 mg/kg	Mazzotti reaction, adverse reaction score twice as high as in ivermectin group; increased punctate opacities starting four days after beginning of treatment, ocular changes resolved by six months after treatment	[29, 30]
Northern Ghana/ controlled RCT with ivermectin versus placebo	17 men; moderate to heavy *O*. *volvulus* infection (~112 mf/mg), 88% with ocular Mf	1,300 mg over eight days, total ~22 mg/kg	Mazzotti reaction; increased punctate corneal opacities starting four days after begin of treatment that were resolved by six months	[52]
Proposed RCTs with IDA versus ivermectin	Ivermectin pretreatment 3 months before IDA. For those with zero Mf in the skin or eye three months later, randomize to treat with IDA or with ivermectin alone at time zero months.	Single IDA treatment contains DEC (6 mg/kg)	Follow-up skin Mf at 6, 12, 18, and 24 months. Adult worms assessed by nodulectomy at 24 months	

*Mazzotti reactions may include fever, pruritus, skin rash, headache, arthralgia, and painful lymphadenopathy.

After many years of DEC use for onchocerciasis, reports indicated that this treatment could cause new ocular lesions or worsen preexisting onchocercal eye disease (Table 1) that included not only the anterior segment of the eye (cornea and anterior chamber) but also the posterior segment (with chorioretinitis and optic nerve atrophy) [18, 25–29]. During the 1980s, a single dose of ivermectin was shown to be more effective than DEC for clearing *O*. *volvulus* Mf from the skin, and ivermectin treatment did not cause ocular adverse events [25, 29–31]. It should be noted that adverse events after DEC treatment correlated with the Mf density in the skin and with the presence of Mf in the eye (cornea, anterior chamber, retina) [32]. A review of ten clinical studies with varying dosing regimens indicated that DEC was usually well tolerated in individuals with low Mf densities of less than 10 Mf/mg skin [16]. However, one study showed that worsening eye disease was associated with the presence of circulating immune complexes in serum prior to and during treatment when patients were treated with high doses and prolonged courses of DEC (150 mg per week for eight weeks, 200 mg per week for four months after an initial course of 1,100 mg over eight days) [33].

Results of clinical and preclinical studies suggest that the host’s immune response is important for DEC and ivermectin efficacy. However, the nature of these interactions is not fully understood [34, 35]. Both drugs have different molecular targets and different dynamics of Mf clearance. After DEC treatment, some *O*. *volvulus* Mf are released into the bloodstream. However, most Mf stay in the skin, where they are attacked by eosinophils and other effector cells and cause a local skin reaction [36]. In contrast, after treatment with ivermectin, Mf are rapidly cleared from the skin and degraded in peripheral lymph nodes [37, 38]. Although both drugs are assumed to have no macrofilaricidal or permanent sterilizing effects on *O*. *volvulus* adult worms, synergistic effects as observed in *W*. *bancrofti* are possible [10, 39]. This hypothesis should be tested with future research.

## The Case for Investigating IDA for Onchocerciasis

Ivermectin is currently widely used for control and elimination of onchocerciasis. A single 150 μg/kg dose usually clears skin Mf and causes temporary sterility for about three to four months, after which Mf newly released from female worms repopulate the skin. The related drug moxidectin (8 μg/kg) clears skin Mf for about twice as long, but the drug is not yet approved for human use [40]. If IDA is as effective against *O*. *volvulus* adult worms as it appears to be against *W*. *bancrofti*, it might permanently sterilize the adult worms and permanently clear Mf from the skin. If it is effective, IDA treatment could also be useful in areas that have had suboptimal responses to ivermectin alone [41].

These considerations strongly support the idea of conducting randomized clinical trials (RCT) to assess the efficacy and adverse event profiles of IDA compared with ivermectin alone in persons with onchocerciasis. The studies will have to be carefully designed to avoid ocular adverse events that have been associated with DEC in the past. However, as mentioned above, the use of single-dose IDA to treat onchocerciasis is fundamentally different from the historical use of DEC therapy for onchocerciasis. Previously, the major goal of DEC treatment was to reduce Mf counts in patients with clinically manifest eye or skin disease and heavy infections by administering DEC for a week or longer. In contrast, the objective of IDA trials in Africa would be to assess IDA’s safety and efficacy as a single-dose regimen for long term clearance of Mf from the skin.

A protocol for clinical trials of IDA in onchocerciasis might include the following features: All participants in the trial should have an ophthalmological assessment prior to enrollment to rule out ocular disease. They should also have Mf present in skin snips and palpable nodules. Participants should all be pretreated with standard dose ivermectin (or ivermectin plus albendazole in areas coendemic for LF). Skin snip testing should be repeated and eye exams should be performed 3 months after treatment. Individuals eligible for the RCT should have no Mf in their eyes by slit lamp examination and zero Mf in skin snips at that time. Participants would then be randomized to receive either ivermectin alone (ivermectin plus albendazole in areas with coendemic LF) or IDA. Participants would be carefully monitored for adverse events by clinicians who are unaware of their treatment assignments. The main efficacy endpoint would be long-term clearance of Mf from the skin as assessed by microscopy and PCR of skin snips. This will be assessed at 6-month intervals for at least 24 months. A secondary endpoint for onchocerciasis IDA studies would be assessment of macrofilaricidal and/or long-lasting embryostatic effects of the treatment by histological examination of adult worms in excised nodules [42]. Safety assessments for IDA in onchocerciasis will include careful monitoring for adverse events following treatment, and this will include repeated ophthalmological examinations.

If RCTs show that IDA is safe and more effective for long-term suppression of Mf than ivermectin alone, the next step would be to perform community trials. Protocol details will need to be developed and carefully reviewed by institutional review boards before studies can be initiated. However, we think it is useful to present a draft outline as a starting point for discussion: Community trial participants would be pretreated with ivermectin or ivermectin plus albendazole and given a “treatment card” to document pretreatment. Three months later, persons with documented pretreatment would be offered treatment with either ivermectin alone or with IDA, and participants would be retested to assess reappearance of Mf in the skin at 12 and 24 months. Persons with Mf in skin snips at 12 months would be retreated with the standard regimen used in their region at that time (ivermectin or ivermectin plus albendazole). The primary endpoints for the study would be percentages of study participants with negative skin snip examinations for Mf at 12 and 24 months. The absence of *O*. *volvulus* DNA in skin snips by PCR would be a useful secondary endpoint.

## Progress of Onchocerciasis Control Sets the Stage for Potential Use of IDA in Africa

The epidemiological situation of onchocerciasis has dramatically changed since the era of widespread DEC use in the 1960s and 70s. Onchocerciasis is believed to be eliminated from most savanna areas in West Africa where blinding onchocerciasis was highly prevalent in the past [43, 44]. Persisting foci of onchocerciasis mainly occur in forested regions of West and Central Africa, where blinding onchocerciasis is not common [45]. Community-directed treatment using ivermectin in hyper- and mesoendemic foci has reduced Mf prevalence and density considerably in the countries of the former African Program for Onchocerciasis Control [46]. However, many of these areas still require ivermectin because of the continuing presence of viable adult female worms that cause low-level microfiladermia (usually less than 5 Mf/mg skin) with little or no eye involvement. These would be suitable areas for community studies of IDA if RCTs have shown that the regimen is safe and effective for treatment of onchocerciasis. The new push for onchocerciasis elimination will also extend mass treatment into hypoendemic areas where Mf rates are less than 20%. The danger of DEC in such areas is likely to be relatively low but not zero because some untreated individuals in hypoendemic areas may have high skin Mf counts with ocular Mf or because they have recently moved from hyperendemic areas. Therefore, documented ivermectin pretreatment at the level of individuals would still be required prior to IDA use in such areas.

## IDA for LF Elimination in Areas Coendemic for Onchocerciasis

**Fig 1:**
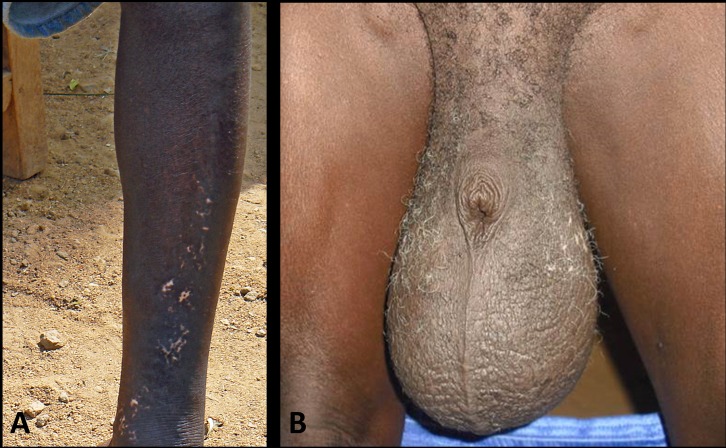
Clinical signs of onchocerciasis (**A**) and lymphatic filariasis (**B**) in the same individual living in an area coendemic for both infections in Lofa County, Liberia. **A** Hypopigmented skin (“leopard skin”) on the shins. **B** Advanced hydrocele.

The proposed IDA RCTs in persons infected with *O*. *volvulus* may serve a dual purpose: First, they may demonstrate that IDA can be safely used for LF elimination in areas that are coendemic for onchocerciasis (Fig 1). Second, they may show that IDA is more effective than ivermectin alone for treatment of onchocerciasis. If preliminary studies show that IDA can be safely used in persons with onchocerciasis following pretreatment with ivermectin, then it potentially could be used in large parts of Africa that are currently using or plan to use ivermectin combined with albendazole for LF elimination. Although we hope that IDA will be superior to ivermectin alone for onchocerciasis, this need not be the case for IDA to be useful for LF elimination programs in areas of Africa with coendemic onchocerciasis.

## Risk and Benefit Considerations of IDA for Onchocerciasis

Carefully designed IDA RCTs in individuals with onchocerciasis will have a low but manageable risk. On the other hand, IDA’s potential benefit is huge because it could significantly accelerate elimination of both LF and onchocerciasis in Africa. It is also possible that IDA will not be safe enough for use as MDA in most regions of Africa. But this possibility should not prevent performance of RCTs for onchocerciasis because a therapy that cannot be used for MDA may still be useful for individual case management. We believe it is likely that a single dose of IDA (administered several months following an Mf-clearing dose of ivermectin) will be much more effective for long-term clearance of *O*. *volvulus* Mf than ivermectin alone. We call this strategy “pretreat and treat.” It is also possible that IDA will kill or permanently sterilize adult female *O*. *volvulus* worms. These hypotheses must be tested because IDA has the potential to completely change the game for onchocerciasis as it is likely to do for LF.

One challenge to be considered early is the risk of uncontrolled use of DEC in countries where it is not currently used because of safety concerns. This should not be a problem for clinical or community trials because DEC would only be used per protocol by trained personnel, and the remaining drug would be removed and destroyed by the research teams. If research studies show that IDA can be safely used with “pretreat and treat” or with other strategies in areas with onchocerciasis, DEC should only be licensed for use by national MDA programs. Any regulatory barriers and restrictions for the use of DEC in onchocerciasis-endemic countries would need to be addressed through the studies and policy change would need to be supported by robust data.

## Evaluation of IDA for Other Filarial Infections in Africa

Of course, *W*. *bancrofti* and *O*. *volvulus* are not the only human filarial parasites that occur in Africa that could be affected by IDA. At present, there is no effective single-dose treatment for *Mansonella perstans*, which may be the most abundant and widespread filarial parasite in Africa [47]. Therefore, it would be important to perform trials to assess the safety of IDA in persons infected with *M*. *perstans*, and these studies would also assess whether IDA is an effective single-dose treatment for this infection. This would be important so that any rollout of IDA for use in LF or onchocerciasis elimination programs would not be limited by the presence of *M*. *perstans*. Because *Mansonella streptocerca* has a limited geographical distribution, usually produces low Mf densities, and is highly susceptible to both DEC and ivermectin, IDA is likely to be safe for use in persons with that infection.

Coendemic loiasis is currently a major barrier to Lf and onchocerciasis elimination in some parts of Africa because ivermectin can lead to serious adverse events including coma and death in patients with high *Loa loa* Mf densities [48]. Neurological serious adverse events have also been described after DEC in subjects with high *L*. *loa* Mf densities [49]. IDA (which contains ivermectin and DEC) is also very likely to be unsafe in individuals with high *L*. *loa* Mf counts. Currently, a test and treat strategy is being evaluated in which individuals are screened for *L*. *loa*, and persons with high blood Mf counts are excluded from MDA with ivermectin [50]. A similar approach could be considered for a clinical trial to explore the value of IDA treatment for individuals with loiasis plus either LF or onchocerciasis. Persons with high *L*. *loa* Mf counts who cannot be treated with ivermectin would be excluded from the study. Persons with lower *L*. *loa* Mf counts could be pretreated with ivermectin alone and later randomized to receive either ivermectin alone or IDA when *L*. *loa* Mf counts are zero or very low. Beyond the safety issue regarding treatment of LF and onchocerciasis in central Africa, IDA (following a clearing dose of ivermectin) has the potential to be the first effective single-dose treatment for loasis, which is itself of clinical significance. Of course, any new antifilarial drug or combination will need to be carefully tested before it can be used in areas that are highly endemic for loiasis.

## Conclusions

IDA has the potential to be a game changer for LF elimination in Africa, especially in countries and regions without coendemic onchocerciasis or loiasis. More research is required to determine if there is a safe and effective way to use IDA in coendemic settings. Additional research will be needed to test whether IDA is safe and effective for treating onchocerciasis, first in individuals and later in endemic populations with or without coendemic LF. We are very enthusiastic about this possibility because IDA is likely to be more effective for clearance and suppression of Mf than ivermectin alone. Compared to drugs that have not yet been tested in humans, IDA provides a potential fast-track and low-cost option that could accelerate elimination of LF and onchocerciasis in Africa.
